# Assessment of independent comorbidities and comorbidity measures in predicting healthcare facility-onset *Clostridioides difficile* infection in Kenya

**DOI:** 10.1371/journal.pgph.0000090

**Published:** 2022-01-31

**Authors:** Winnie C. Mutai, Marianne Mureithi, Omu Anzala, Brian Kullin, Robert Ofwete, Cecilia Kyany’ a, Erick Odoyo, Lillian Musila, Gunturu Revathi

**Affiliations:** 1 Department of Medical Microbiology, School of Medicine, University of Nairobi, Nairobi, Kenya; 2 Division of Medical Virology, Department of Pathology, Faculty of Health Sciences, University of Cape Town, Cape Town, South Africa; 3 US Army Medical Research Directorate-Africa, Kenya, Nairobi, Kenya; 4 Department of Pathology, Division of Medical Microbiology, Aga Khan University Hospital, Nairobi, Kenya; University of Bath, UNITED KINGDOM

## Abstract

**Introduction:**

*Clostridioides difficile* is primarily associated with hospital-acquired diarrhoea. The disease burden is aggravated in patients with comorbidities due to increased likelihood of polypharmacy, extended hospital stays and compromised immunity. The study aimed to investigate comorbidity predictors of healthcare facility-onset *C*. *difficile* infection (HO-CDI) in hospitalized patients.

**Methodology:**

We performed a cross sectional study of 333 patients who developed diarrhoea during hospitalization. The patients were tested for CDI. Data on demographics, admission information, medication exposure and comorbidities were collected. The comorbidities were also categorised according to Charlson Comorbidity Index (CCI) and Elixhauser Comorbidity Index (ECI). Comorbidity predictors of HO-CDI were identified using multiple logistic regression analysis.

**Results:**

Overall, 230/333 (69%) patients had comorbidities, with the highest proportion being in patients aged over 60 years. Among the patients diagnosed with HO-CDI, 63/71(88.7%) reported comorbidities. Pairwise comparison between HO-CDI patients and comparison group revealed significant differences in hypertension, anemia, tuberculosis, diabetes, chronic kidney disease and chronic obstructive pulmonary disease. In the multiple logistic regression model significant predictors were chronic obstructive pulmonary disease (odds ratio [OR], 9.51; 95% confidence interval [CI], 1.8–50.1), diabetes (OR, 3.56; 95% CI, 1.11–11.38), chronic kidney disease (OR, 3.88; 95% CI, 1.57–9.62), anemia (OR, 3.67; 95% CI, 1.61–8.34) and hypertension (OR, 2.47; 95% CI, 1.–6.07). Among the comorbidity scores, CCI score of 2 (OR 6.67; 95% CI, 2.07–21.48), and ECI scores of 1 (OR, 4.07; 95% CI, 1.72–9.65), 2 (OR 2.86; 95% CI, 1.03–7.89), and ≥ 3 (OR, 4.87; 95% CI, 1.40–16.92) were significantly associated with higher odds of developing HO-CDI.

**Conclusion:**

Chronic obstructive pulmonary disease, chronic kidney disease, anemia, diabetes, and hypertension were associated with an increased risk of developing HO-CDI. Besides, ECI proved to be a better predictor for HO-CDI. Therefore, it is imperative that hospitals should capitalize on targeted preventive approaches in patients with these underlying conditions to reduce the risk of developing HO-CDI and limit potential exposure to other patients.

## Introduction

*Clostridioides difficile* is a significant nosocomial pathogen contributing to approximately 12% of health care facility-associated diarrhoea in the USA [1]. It is well known that *C*. *difficile* forms part of the diverse microbiota in the gut. Nevertheless, gut changes that cause a reduction in the gut microbial diversity potentiate the overgrowth and establishment of pathogenic *C*. *difficile*. While antibiotic exposure is typically a prerequisite for *C*. *difficile* infection (CDI), epidemiological evidence especially from developed countries has established that advanced age, extended hospital length of stay, comorbidities and the use of acid-suppressive agents are additional predictors implicated in the development of healthcare facility-onset *C*. *difficile* infection (HO- CDI) [2].

Despite the investigation of these key risk factors, cases of CDI continue to rise. Furthermore, while the significance of comorbidities on CDI has been well established in developed countries, limited research from resource-limited countries is available. Previous studies have attempted to describe comorbidities commonly associated with HO-CDI and they have shown that chronic kidney disease, HIV/AIDS and other immunodeficiency disorders, chronic obstructive pulmonary disease, inflammatory bowel disease, hematological malignancy, and diabetes mellitus increase the risk of both initial and recurring CDI [3–6]. Other comorbidities that have been implicated include cardiovascular disease, digestive disorders, dementia, cerebrovascular disease, congestive heart failure, peripheral vascular disease, and myocardial infarction [7, 8]. However, the actual pathophysiological mechanisms and specific relationship of how these comorbidities influence the development of CDI is not well understood [6]. Certainly, comorbidities are known to down-regulate the immune system and cause organ dysfunction. However, patients with these comorbidities are more likely to be hospitalized and receive antibiotics, which places them at an increased risk of HO-CDI.

It is important to evaluate the effect of individual comorbidities on HO-CDI, as most of these comorbid conditions are interrelated and therefore the predictions may be overestimated. As such, the concept of stratification of comorbidities using validated comorbidity indices or aggregated scores minimizes the effect of correlation while still controlling for potential confounding variables. The Charlson Comorbidity Index (CCI) and Elixhauser Comorbidity Index (ECI) are widely used measures in health research to access comorbidities and have previously been applied in studies to predict the risk of CDI. While CCI measures 19 comorbid conditions weighted 1 to 6, ECI has a more extensive list of 31 conditions considering additional conditions such as hypertension, weight loss, obesity and psychiatric disorders that are excluded from other indices. Higher Charlson and Elixhauser comorbidity scores have previously been correlated with CDI and represent an increased likelihood of developing HO-CDI [5, 7, 8].

Until recently, studies in Kenya have demonstrated the existence of CDI in hospitalized patients; however, none of them have evaluated the comorbidities defined by the *International Classification of Diseases*, *Tenth Revision*, *Clinical Modification* (ICD-10-CM) as potential risk factors for HO-CDI [9–11]. To build on existing knowledge and generate data from developing countries, we assessed comorbidities in a population previously tested for HO-CDI. The information may help in stratifying patients with significant comorbidities facilitating the design of prevention approaches and targeted treatment at an early stage of HO-CDI diagnosis.

## Methodology

### Data collection

#### Study population

Using a cross sectional study approach, we enrolled 333 hospitalized patients between 2016–2018 [9]. The inclusion criteria comprised of all age groups who developed diarrhoea > 3days after admission. Data were obtained by conducting interviews with adult patients or guardians of minors and reviewing their files to check for consistency and additional information. A data collection form was used to collect information on age, gender, admission ward, duration of hospitalization, diagnosis on admission, previous history of admission, medication used, and existing comorbidities. Comorbidities reported by the patients or indicated in the patient files were used to calculate CCI and ECI scores.

#### Outcome variable

The study outcome variable was healthcare facility-onset *C*. *difficile* infection (HO-CDI). HO- CDI was defined as the onset of diarrhoea >3 days after admission to a healthcare facility and a positive result for amplification of the *C*. *difficile tpi* gene, combined with one or more toxin genes (*tcdA*, *tcdB*, *cdtA*/*cdtB*) based on a previously described nucleic acid amplification test [9].

#### Predictor variables and confounders

The study investigated individual comorbid conditions defined by the *International Classification of Diseases*, *Tenth Revision*, *Clinical Modification* (ICD-10-CM). Comorbidity was defined as the pre-existence of one or more medical conditions coexisting with the primary condition [12]. A total of 22 specific comorbidities were considered: congestive heart failure, cardiac arrhythmias, chronic obstructive pulmonary disease (COPD), hypertension, peptic ulcer disease, diabetes, hemiplegia, hypothyroidism, pulmonary circulation disorders, chronic kidney disease (CKD), liver disease, solid tumor without metastasis, metastatic solid tumor, HIV/AIDS, lymphoma, weight loss (malnutrition), anemia, and depression. Each of these individual comorbidities was investigated separately. To construct comorbidity scores, each comorbid condition was assigned a weight based on the relative risk of mortality for risk adjustment (S1 Table). Then the indexes were summed-up to provide the total scores and categories before exploring their association with HO-CDI.

The potential confounders included age, hospitalization duration, medication administered (antibiotics, laxatives, analgesics, antiretrovirals, chemotherapy agents), previous admission, primary disease (a condition present at admission), gastrointestinal procedures (colonoscopy/endoscopy and surgery).

### Ethical approval

This study was approved by the Kenyatta National Hospital-University of Nairobi Ethics and Research Committee (P8/01/2014). Written informed consent was obtained from adult participants and legal guardians of the minors.

### Statistical analysis

Descriptive statistics for demographic and clinical information of the study participants were computed and the outcomes were expressed as frequencies and percentages and summarized in Table 1. Individual comorbidities and comorbidity indices were profiled in Table 2 where z test for proportion was used to test for significant differences in individual comorbidities and Pearson Chi-Square applied to test for association between CCI and ECI groups and HO-CDI outcome. A binary logistic regression analysis was conducted in a sequential approach to identify significant comorbidity predictors of HO-CDI and presented in a forest plot.

**Table 1:** Baseline characteristics of the study participants.

Variable	With comorbidity (n = 230)	Without comorbidity (n = 103)	Total
Age (years)			
≤ 5	95 (78.5)	26 (21.5)	121 (100)
6–15	18 (75)	6 (25)	24 (100)
16–25	13 (41.9)	18 (58.1)	31 (100)
26–45	61 (56.5)	47 (43.5)	108 (100)
46–59	26 (86.7)	4 (13.3)	30 (100)
≥ 60	17 (89.5)	2 (10.5)	19 (100)
Gender			
Female	117 (68.8)	53 (31.2)	170 (100)
Male	113 (69.3)	50 (30.7)	163 (100)
Duration of hospitalization			
≤30days	159 (70)	68 (30)	227 (100)
>30days	71 (70.3)	35 (34.7)	101 (100)
Medication exposure			
Antibiotics	221 (74.4)	76 (25.6)	297 (100)
Acid suppressive agents	85 (82.5)	18 (17.5)	103 (100)
Antiretrovirals	49 (96.1)	2 (3.9)	51 (100)
Chemotherapy treatment	11 (73.3)	4 (26.7)	15 (100)
Laxatives	12 (70.6)	5 (29.4)	17 (100)
Analgesic	44 (56.4)	34 (43.6)	78 (100)
Diagnosed with HO-CDI			
Yes	63 (88.7)	8 (11.3)	71 (100)
No	167 (63.7)	95 (36.3)	262 (100)
Previous hospital admission (prior 3 months)			
Yes	44 (91.7)	4 (8.3)	48 (100)
No	186 (65.3)	99 (34.7)	285 (100)

HO-CDI, healthcare facility-onset *C*. *difficile* infection.

**Table 2:** Pairwise comparison of HO-CDI outcome to individual comorbidity conditions and comorbidity indices.

Comorbidity	Patients with HO-CDI (n = 71) (%)	Patients without HO-CDI (n = 262) (%)	*P*
Congestive heart failure	1 (1.4)	1(0.4)	ND
Chronic obstructive pulmonary disease	7 (9.9)	4 (1.5)	<0.001
Peptic ulcer disease	8 (11.3)	14 (5.3)	0.075
Peripheral vascular disease	0 (0)	1 (0.4)	ND
Liver disease	2 (2.8)	3 (1.2)	0.304
Diabetes	12 (16.9)	18 (6.9)	0.009
Hemiplegia or paraplegia	0 (0)	3 (1.1)	ND
Chronic kidney disease	11 (15.5)	12 (4.6)	0.001
Leukemia	4 (5.6)	0 (0)	ND
Metastatic solid tumor	2 (2.8)	0 (0)	ND
HIV/AIDS	18 (25.4)	41 (15.7)	0.058
Cardiac arrhythmias	1 (1.41)	0 (0)	ND
Hypertension	17 (23.9)	25 (9.5)	0.001
Hypothyroidism	1 (1.4)	0 (0)	ND
Lymphoma	2 (2.8)	0 (0)	ND
Solid tumor without metastasis	4 (5.6)	5 (1.9)	0.086
Weight loss (Malnutrition)	7 (9.9)	28 (10.7)	0.840
Anemia	16 (22.5)	25 (9.5)	0.003
Depression	0 (0)	1 (0.38)	ND
Tuberculosis	15 (21.1)	25 (9.5)	0.008
Rickets	1 (1.4)	19 (7.3)	0.066
Inflammatory bowel disease	2 (2.8)	6 (2.3)	0.797
**Charlson comorbidity scores**			<0.001*
CCI = 0	33 (46.5)	197 (74.4)	
CCI = 1	4 (5.6)	8 (3.1)	
CCI = 2	11 (15.5)	14 (5.3)	
CCI ≥3	23 (32.4)	43 (16.4)	
**Elixhauser comorbidity scores**			<0.001*
ECI = 0	16 (22.5)	142 (54.2)	
ECI = 1	28 (39.4)	72 (27.5)	
ECI = 2	15 (21.1)	35 (13.4)	
ECI ≥3	12 (16.9)	13 (5)	

CCI, Charlson Comorbidity Index; ECI, Elixhauser Comorbidity Index

* Pearson Chi-Square test was conducted to assess the relationship between CCI and ECI comorbidity scores and the outcome of HO-CDI.

**Fig 1:**
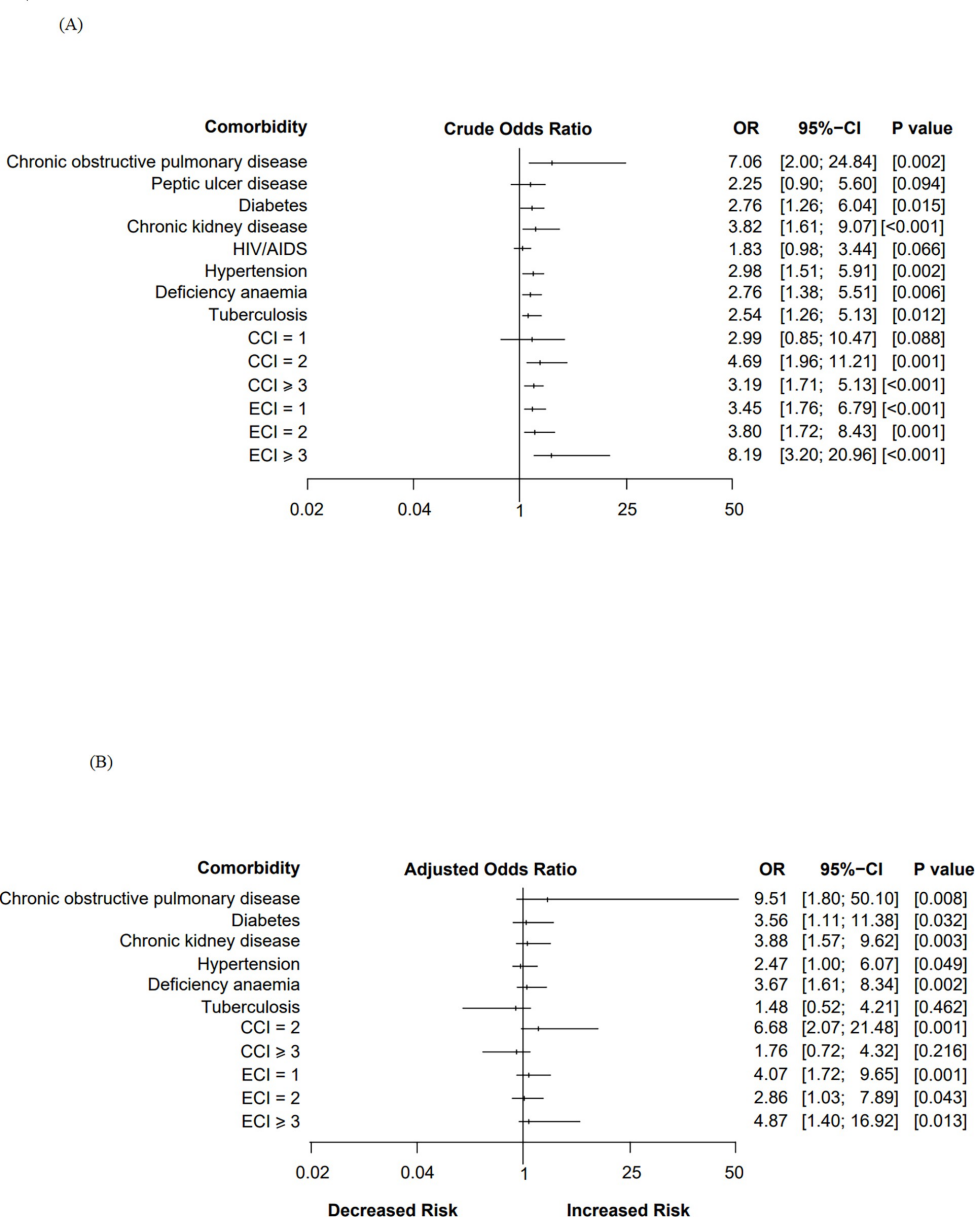
Forest plot depicting odds ratios (OR) with 95% confidence interval (95% CI) of predicting HO-CDI in patients with different comorbidity profiles. (A) Univariate logistic regression model showing unadjusted crude odds ratios. (B) Multiple binary logistic regression model showing adjusted odds ratios. The horizontal lines indicate the width of the confidence interval while the vertical marks on each horizontal line show the odds ratios. An odds ratio of more than 1.0 indicates increased risk. Abbreviations: OR- Odds ratio; CI- Confidence Interval; CCI, Charlson Comorbidity Index; ECI, Elixhauser Comorbidity Index; *p*-value indicating the level of statistical significance (*p* < 0.05).

First, variables with *p*-values of ≤ 0.2 from Table 2 and those known to be clinically relevant based on literature were selected for the model development stage. The variables were individually fitted into a bivariate binary logistic regression model to obtain crude odds ratios of comorbidities/comorbidity scores associated with the likelihood of developing HO-CDI (Fig 1A. Secondly, variables whose *p*-values were ≤0.05 in the bivariate analyses were considered in a final multiple binary logistic regression model where potential confounders were controlled (Fig 1B). Adjusted odds ratio (AOR), corresponding *p*-value and the 95% confidence interval (CI) were used to identify significant independent comorbidities and comorbidity scores associated with the risk of developing HO-CDI. Variables with *p* ≤ 0.05 were considered statistically significant. Likelihood ratio test was used to assess for goodness-of-fit that is whether adding more parameters to ECI and CCI models had significant impact in predicting the outcome of CDI. Here, the likelihood ratio test static (assumed to follow chi-squared distribution) was generated by getting the difference between log-likelihoods of the simple and complex models, and degrees of freedom represented by additional parameters in the complex model. Finally, to determine the CCI and ECI performance in predicting HO-CDI outcome, the model fit was assessed using a pseudo-R squared where the model with the higher value was considered a better predictor of HO-CDI.

For each variable, patients without the individual comorbid conditions were the reference group while for comorbidity scores, a score of 0 was the reference value in the analysis. The statistical analysis and visualization were performed using STATA version 13.1.

## Results

### Demographic and participants characteristics

The baseline characteristics of the 333 patients with and without comorbidities are summarized in Table 1. A total of 230/333 (69%) had comorbidities, while 102/333 (31%) did not report any history of comorbidities. The proportion of comorbidities was greater in patients aged over 60 years. Additionally, higher proportions of medication exposure were recorded in the group with comorbidities: antiretrovirals 49/51 (96.1%), acid-suppressive agents 85/103 (82.5%) and antibiotics 221/297 (74.4%) than in the non-comorbid group. Among the total number of patients who reported prior hospital admission in the preceding three months, 44/48 (91.7%) had comorbid conditions while those diagnosed with HO-CDI were 63/71 (88.7%). However, there were no proportional differences in the duration of hospitalization (≤30 or >30 days) in the comorbid and non-comorbid groups.

Independent comorbidities that differed significantly between the HO-CDI patients and the comparison group included hypertension (23.5% vs. 9.5%), anemia (22.5% vs. 9.5%), tuberculosis (21.1% vs. 9.5%), diabetes (16.9% vs. 6.9%), chronic kidney disease (15.5% vs. 4.6%) and chronic obstructive pulmonary disease (9.9% vs. 1.5%). Additionally, the results revealed that although there were more patients with HIV/AIDS (25.4% vs. 15.7%) and peptic ulcer disease (11.3% vs. 5.3%) in the HO-CDI group, the difference in proportions were not significant. A majority of the participants had a score of zero in both the CCI (*n* = 230) and ECI (*n* = 158). However, more than a third of the participants who had a CCI score of 1 (33.3%), 2 (44%) and ≥ 3 (34.8%) were positive for HO-CDI compared to only 14.4% who had a score of 0. Also, 28%, 30% and 48% of the participants positive for HO-CDI had an ECI score of 1, 2, and ≥ 3, respectively. The differences between the categories of both the CCI and ECI were statistically significant at *p* < 0.001. Summary statistics comparing the proportions of HO-CDI outcome by individual comorbidities and comorbidity scores are illustrated in Table 2.

Independent comorbidities including congestive heart failure, peripheral vascular disease, hemiplegia, leukemia, metastatic solid tumor, cardiac arrhythmias, hypothyroidism, lymphoma and depression were not explored further because of their relatively low frequencies.

### Logistic regression analysis of potential comorbidity predictors of HO-CDI

The unadjusted and adjusted logistic regression models were analyzed to explore the association between comorbidities and HO-CDI (Fig 1A and 1B). Eight individual comorbidities with *p*-values of ≤ 0.2 and all the comorbidity scores in each category were individually fitted into a crude logistic regression model. Following univariate analysis, patients with chronic pulmonary disease (Odds ratio, OR, 7.06; 95% Confidence interval, CI, 2–24.84; *p* < 0.05), chronic kidney disease (OR, 3.82; 95% CI, 1.61–9.04; *p* < 0.001), hypertension (OR, 2.98; 95% CI, 1.51–5.91; *p* < 0.05), diabetes (OR, 2.76; 95% CI, 1.26–6.04; p < 0.05), anemia (OR, 2.76; 95% CI, 1.38–5.51; *p* < 0.05) and tuberculosis (OR, 2.54; 95% CI, 1.26–5.13; *p* < 0.05) were more likely to have HO-CDI conditions compared to non-HO-CDI patients. Additionally, patients with CCI scores of 2 (OR, 4.69; 95% CI, 1.96–11.21; *p* < 0.001) and ≥ 3 (OR, 3.19; 95% CI, 1.71–5.97; *p* < 0.001) were more likely to develop HO-CDI as were those with ECI scores of 1, 2 and ≥ 3. Peptic ulcer disease and HIV/AIDS were not significant in the crude logistic regression model and were therefore not fitted into the adjusted logistic regression model.

After adjusting for all the potential confounders, five independent comorbidities were identified as potential predictors of HO-CDI: chronic obstructive pulmonary disease (OR, 9.51; 95% CI, 1.80–50.1), diabetes (OR, 3.56; 95% CI, 1.11–11.384), chronic kidney disease (OR, 3.88; 95% CI, 1.57–9.62), anemia (OR, 3.67; 95% CI, 1.61–8.34) and hypertension (OR, 2.47; 95% CI, 1–6.07). In comparison to patients who did not have tuberculosis, patients with tuberculosis were 48% more likely to develop HO-CDI although this was not statistically significant. In reference to comorbidity scores, while adjusting for all confounding variables and other comorbidities, the patients who had a CCI score of 2 were 6.67-times (95% CI: 2.07–21.48; *p* < 0.001) more likely to have HO-CDI compared to patients who did not have CCI comorbidities (i.e., CCI = 0), while patients who had an ECI score of 1, 2 and ≥ 3 were associated with a 4.07-times (95% CI: 1.72–9.65; *p* < 0.001), 2.86-times (95% CI: 1.03–7.89; *p* < 0.05) and 4.87-times (95% CI: 1.40–16.92; *p* < 0.05) increased odds of HO-CDI, respectively.

## Discussion

This study is the first to assess independent comorbidities and comorbidity scores that increase the risk of developing HO-CDI in hospitalized patients in a Kenyan cohort. We observed that the majority of the hospitalized patients had underlying conditions with significantly higher proportions in the older population. In addition, an overall significantly higher rate of HO-CDI was observed in the patient population with comorbidities.

Comorbidities with higher prevalence in patients with HO-CDI included HIV/AIDS, followed by hypertension, anemia, tuberculosis, diabetes, chronic kidney disease, peptic ulcer disease and chronic obstructive pulmonary disease. Most of these comorbidities would necessitate polypharmacy and prolonged hospital admission directly influencing the shift from *C*. *difficile* colonization to subsequent CDI [13]. Consistent with previous studies, independent comorbidities including hypertension, chronic kidney disease, anemia, diabetes, chronic obstructive pulmonary disease as well as aggregate Charlson Comorbidity scores and Elixhauser Comorbidity scores were significantly associated with increased risk of HO-CDI [5, 8, 14].

In the present study we noted that chronic diseases including diabetes, hypertension, chronic kidney disease and chronic obstructive pulmonary disease were significantly highly ranked predictors of HO-CDI. It is known that a chronic disease naturally compromises the immune system. Consequently, decreased immunological tolerance among our study participants would have increased their susceptibility to infections, likely leading to more antibiotic intake and prolonged hospitalization. Moreover, the descriptive statistics from this study showed that antibiotics and previous history of admission, which are both potential risk factors of developing HO-CDI were significantly higher among the patients with comorbidities. Interestingly, even after adjusting for these factors in the multiple logistic regression, strong correlations were still observed. Thus, our results suggest that chronic diseases are significant predictors of HO-CDI as noted earlier [15–17].

Findings from this study suggest that COPD was among the significant predictors resulting in 9.5 times increased risk of HO-CDI. A possible explanation for this could be that the increased susceptibility to bacterial respiratory tract infections in COPD patients contributes to greater consumption of antibiotics, which in turn predisposes patients to HO-CDI [18]. According to Jasiak et al, COPD resulted in a 3.5-fold increased risk of recurrent HO-CDI [19].

Previous studies comparing patients with and without underlying chronic kidney disease (CKD) noted that the former had a higher risk of initial and recurrent episodes of CDI [20, 21]. Similarly, these findings were supported by a recent study that observed an almost four-fold increased risk (OR:3.68, CI: 1.63–8.31, p = 0.002) of developing CDI in patients with underlying CKD [22]. The reduced function of the kidney not only interferes with the elimination of the toxins from the body but also alters the functions of the intestinal microbiota and activates systemic inflammation [23–25]. These observations could therefore explain the increased susceptibility of HO-CDI in patients with CKD.

Hypertension was the highest comorbidity observed among the study participants and was significantly associated with an increased risk of developing HO-CDI. Previous investigations have reported similar findings [26, 27]. Currently, the reason behind the increased risk is not apparent, however, accumulating evidence using both animal and human models suggests that hypertension influences gut microbiota dysbiosis [28, 29]. On the other hand, antihypertensive drugs have been shown to improve or compromise intestinal microbiota [30, 31]. Verapamil, for example, protected the cells from *C*. *difficile* intoxication [32]. We however did not collect any information on antihypertensive medication in this study. Therefore, based on the data we could not ascertain whether hypertension itself or the hypertensive medication was responsible for increased odds of developing HO-CDI.

Another important chronic disease predictor observed in this study was diabetes. Patients with diabetes were three times more likely to develop CDI compared to non-diabetic patients. The relationship between CDI and diabetes has been studied extensively. Diabetes has been established as a possible independent risk factor for primary and recurrent CDI [33–35]. Diabetes causes structural remodeling of the colon that affects various functions of the gastrointestinal tract leading to, amongst other things, impaired motility and an altered composition of the intestinal microbiota, which may contribute to *C*. *difficile* driven diarrhoea [36, 37]. On the other hand, in their case-control study Eliakim-Raz et al. observed that diabetic patients treated with metformin an anti-diabetic drug had reduced odds (OR 0.58; 95% CI, 0.37–0.93; p = 0.023) of developing CDI compared to their counterparts [38]. Similarly, an interventional study observed that metformin-treated diabetic patients had a reduced abundance of *Clostridium* spp., which could significantly impact *C*. *difficile* colonization [39]. Although the exact mechanism behind this is not clear, a potential mechanism that has been investigated is that metformin alters the reabsorption of secondary bile acids and as a result inhibits spore germination, vegetative growth and toxin activity of *C*. *difficile* strains [40–42]. Therefore, even though a causal relationship has not been established, it is evident that structural and functional changes in the colon induced by diabetes itself or diabetes medication are likely to alter the composition of the gut microbiota, which consequently increases or reduces the risk of CDI [43].

Univariate analysis showed an association between tuberculosis and HO-CDI, however after adjusting for potential confounders including anti-tuberculosis treatment, no statistical difference was observed in patients with HO-CDI in comparison with patients without HO-CDI. Thus, the relationship between tuberculosis and HO-CDI may have occurred because of the confounding effect of anti-tuberculosis drug exposure. Rifampicin was previously shown to induce CDI in patients receiving anti-tuberculosis treatment [44]. Additionally, prolonged use of rifampicin has resulted in high resistance rates in some settings, consequently promoting the persistence of resistant *C*. *difficile* strains in patients undergoing tuberculosis treatment [45, 46]. In support of this, we previously reported that a large proportion of *C*. *difficile* strains isolated from the same study population showed resistance to rifampicin [9].

Although HIV/AIDS was a frequent comorbidity, this group of patients had 42% lower odds of developing HO-CDI. However, we noted that a majority of these patients were receiving concomitant antiretroviral therapy and consequently this would have an effect in reducing the risk of CDI as previously described [47]. In addition, future studies should provide more insights on the risk of developing HO-CDI in patients with HIV/AIDS as some studies have suggested a possible association between pre-existing HIV/AIDS and CDI in both adults and children [48, 49].

The present study failed to establish a correlation between HO-CDI and underlying peptic ulcer disease, liver disease, inflammatory bowel disease, low levels of vitamin D (rickets), solid tumor without metastasis, and weight loss (malnutrition) as previously described [17, 50–54]. Future clinical studies should explore these associations considering the possibility of increased antibiotic use and hospital admission.

In both CCI and ECI classification, there was sufficient evidence (*p* < 0.001) to reject the null hypothesis and conclude that there is an association between the CCI and ECI comorbidity scores and the primary outcome of HO-CDI. Despite their differences in weighting and number of comorbidities, both models performed well with minor differences in their validation values. It is interesting that patients with CCI ≥3 were no more likely to have HO-CDI than those with scores of 0. A possible reason for this might be that patients in this group are regarded as having moderate and severe comorbidity levels raising the likelihood that their diarrhoea is due to causes other than CDI. In testing for goodness-of-fit, the adjusted/complex model was shown to fit the dataset significantly better (p value < 0.0001) for both the CCI and ECI groupings. However, most remarkable observation from the analysis was that the Elixhauser classification emerged as a better predictor than the Charlson classification in both the unadjusted (Pseudo R-squared 7.89 vs 6.09) and adjusted models (Pseudo R-squared 27.55 vs 27.04). These findings are consistent with previous studies where the Elixhauser grouping was reported to be a better predictor of an outcome while compared to the Charlson grouping, albeit by a small margin [55].

Although solid evidence linking comorbidities with HO-CDI was observed, this study, however, had some limitations. First, the study participants were enrolled from a single centre and hence the findings may not be generalized to other healthcare facilities within the country and therefore future studies should consider a multicentre approach. Secondly, data collection relied mostly on what was indicated in each patient’s file, which could have contributed to underreporting of some conditions. Finally, for some conditions like diabetes, it was not classified as complicated or uncomplicated as required by ICD-10-CM.

In summary, chronic obstructive pulmonary disease, chronic kidney disease, anemia, diabetes, and hypertension were significant predictors of HO-CDI in our setting. Therefore, it is recommended that patients with these co-morbidities be identified early and, where possible, procedures implemented that serve to limit potential exposure to other patients with CDI and/or environments likely to be contaminated by spores. Interventions aimed at restoring and maintaining the resident gut microbiota may also be beneficial in this patient population. The study also suggests that stratification of comorbidities according to ECI rather than CCI would further help to identify at-risk patients since it was a better predictor for HO-CDI.

## Supporting information

S1 Table Assigned weights of Charlson and Elixhauser Comorbidity Index. (DOCX)

S1 Data Data supporting information file. (XLS)
