# Supplementary material for: Assessment of independent comorbidities and comorbidity measures in predicting healthcare facility-onset Clostridioides difficile infection in Kenya
Source: PLOS Glob Public Health. 2022 Jan 31;2(1):e0000090. doi: 10.1371/journal.pgph.0000090 (PMC10022263; doi:10.1371/journal.pgph.0000090)
Supplement: S1 Table — (DOCX) [file pgph.0000090.s001.docx]

S1 Table. Assigned weights of Charlson and Elixhauser Comorbidity Index.

|  | **Charlson Comorbidity Index (CCI)** | **Elixhauser Comorbidity Index (ECI)** |
| --- | --- | --- |
| **Condition^a^** | **Assigned weight** | **Assigned weight^b^** |
| Congestive heart failure | 1 | 7 |
| Cardiac arrhythmias | - | 5 |
| Chronic obstructive pulmonary disease | 1 | 3 |
| Peripheral vascular disease | 1 | 2 |
| Hypertension | - | 0 |
| Diabetes | 2 | 0 |
| Peptic ulcer disease | 1 | 0 |
| Hemiplegia | 2 | 7 |
| Hypothyroidism | - | 0 |
| Renal disease | 2 | 5 |
| Liver disease | 3 | 11 |
| Solid tumor without metastasis | 2 | 4 |
| Metastatic solid tumor | 6 | 12 |
| HIV/AIDS | 6 | 0 |
| Lymphoma | 2 | 9 |
| Leukaemia | 2 | - |
| Weight loss (malnutrition) | - | 6 |
| Anemia | - | -2 |
| Depression | - | -3 |
| Tuberculosis | - | - |
| Rickets | - | - |
| Inflammatory bowel disease | - | - |

^a^, The comorbidities ICD-10 codes; ^b^ van Walraven's weights.
